# On Diabetes and Its Successful Treatment

**Published:** 1861-03

**Authors:** 


ART. VII.—*On Diabetes and its Successful Treatment.* By JOHN M. CAMPLIN, M.D., F.L.S. 12mo., pp. 87. New York: S. S. & W. Wood, 1861.

THIS useful brochure, a reprint from the second London edition, originally appeared in 1858, as a republication of a paper from the Transactions of the Royal Medical and Chirurgical Society, and under its new garb is now brought to our notice, with additions having reference to the physiology of the organs concerned in this complicated disease, the value of iodine and pepsin in its treatment, and cases illustrative of its medical management.

Dr. Camplin devotes a few pages of this essay to the consideration of the pathology of diabetes, considering it as an affection in which the nervous system is more or less at fault, and the nervous power materially exhausted, and inferring that the brain and nervous system are primarily affected. He does not concur in the opinion of M. Bernard and other eminent physiologists, that the formation of sugar is alone referrable to the liver, but says:—

"For many years I have been accustomed to consider the views of Dr. Prout on the pathology of diabetes as correct, with reference to the stomach and primæ viæ, and that the change of amylaceous matters into sugar takes place in that part of the system; and notwithstanding the recent researches, which give the glucogenic function to the liver, I am disposed to believe that in many cases the gastric juice may be so changed as to resemble the saliva in its properties, and that sugar may be absorbed independently of the liver."

Passing by the theories of the production of the disease, we come to the more important part of the work, and will briefly consider the juvantia and lædentia in diabetes, premising by stating that the observations of Dr. Camplin were made in the first place upon his own person, and that the successful management of his own case, as well as the experience of other physicians, have fully confirmed the soundness of the principles which he has laid down. The diet should be perfectly plain and nourishing, and all amylaceous articles be withheld. The patient should be invigorated with tonics, the action of the skin should be promoted, and the action of the liver and primæ viæ be regulated. Cold and damp are to be avoided, and in winter the patient should be warmly clad. All varieties of animal food and fish may be taken with impunity, as is the case also with milk and eggs. In regard to vegetables, onions, turnips, pickles, and lettuces properly dressed, are important. Young cabbage is considered by the author to be preferable to all other cruciferæ in the actually diabetic, and cauliflower sprouts, sea-kale, celery, mushrooms, beans, and endive will make a pleasing variety. As a beverage, good French brandy, claret, or Bordeaux wine are the best articles, all other wines and malt liquors being avoided. Tea will be found to be better than coffee, although the patient may take either. In persons disposed to obesity with a feeble circulation, animal food, with vegetables and fruits, should be used rather than the amylaceous articles; but fruits are, in general, not to be employed. Dr. Camplin places more confidence in bran cake than in all other articles of diet, and to show how efficaciously a diet of animal food, vegetables, and bran cake acts, we may state that the author suffered under a return of the disease from a change of diet, the urine being of high specific gravity and containing seventeen grains of sugar to the ounce. Four days after a return to the above diet, the urine was found to contain less than half a grain of sugar. Bran cakes being in every case a most important adjuvant, we give the formula for making them :—

"*Formula for Bran Cakes.*—Take a sufficient quantity (say a quart) of wheat bran, boil it in two successive waters for a quarter of an hour, each time straining it through a sieve, then wash it well with cold water (on the sieve) until the water runs off perfectly clear; squeeze the bran in a cloth as dry as you can, then spread it thinly on a dish, and place it in a slow oven; if put in at night, let it remain until the morning, when, if perfectly dry and crisp, it will be fit for grinding. The bran thus prepared must be ground in a fine mill, and sifted through a wire sieve of such fineness as to require the use of a brush to pass it through; that which remains in the sieve must be ground again, until it becomes quite soft and fine.* Take of this bran powder three ounces, (some patients use four ounces, the other ingredients as follows,) three new-laid eggs, one and a half (or two ounces if desired) of butter, and about half a pint of milk; mix the

* This is particularly necessary in cases of irritable bowels.

eggs with a little of the milk, and warm the butter with the other portion; then stir the whole well together, adding a little nutmeg and ginger, or any other agreeable spice. Bake in small tins, (pattipans,) which must be well buttered, in a rather quick oven, for about half an hour. The cakes, when baked, should be a little thicker than a captain's biscuit; they may be eaten with meat or cheese, for breakfast, dinner, and supper; at tea they require rather a free allowance of butter, or may be eaten with curd or any of the soft cheeses. * * *

"In some seasons of the year, or if the cake has not been well prepared, it changes more speedily than is convenient; this may be prevented by placing the cake before the fire for five or ten minutes every day."

There is no specific remedy for this disease, but Dr. Camplin has great confidence in the effervescing citrate of ammonia, in combination generally with nitrate of iron. Bitters and alkalies will be found serviceable in most cases, and cod-liver oil, when indicated, combined with wine of iron or infusion of quassia, may be administered. Opium may be given to check temporarily the flow of urine when unusually great, and in younger subjects the mineral acids, especially the nitro-muriatic, or hydrochloric in combination with iron, or the tincture of the chloride, will be useful. Every case, however, presenting certain modifications, the treatment must be varied in accordance with the constitution of the patient, or the organs concerned in the attack. Pepsin has not been extensively tried, but in some cases has produced remarkable effects. From the fact of iodine checking diuresis in horses, the iodides may prove serviceable in the human subject, and the iodide of iron will probably be the best form to employ.

Finally, the diabetic diet, consisting of the bran cake, milk, eggs, all kinds of animal food and fish, greens and other vegetables, to effect a cure or produce even temporary relief, must be persisted in, and the patient must not indulge in fruits or other amylaceous articles.

